# Genotypic Diversity Is Associated with Clinical Outcome and Phenotype in Cryptococcal Meningitis across Southern Africa

**DOI:** 10.1371/journal.pntd.0003847

**Published:** 2015-06-25

**Authors:** Mathew A. Beale, Wilber Sabiiti, Emma J. Robertson, Karen M. Fuentes-Cabrejo, Simon J. O’Hanlon, Joseph N. Jarvis, Angela Loyse, Graeme Meintjes, Thomas S. Harrison, Robin C. May, Matthew C. Fisher, Tihana Bicanic

**Affiliations:** 1 Institute of Infection and Immunity, St. George’s University London, London, United Kingdom; 2 Department of Infectious Disease Epidemiology, Imperial College School of Public Health, London, United Kingdom; 3 School of Medicine University of St. Andrews, St. Andrews, United Kingdom; 4 Botswana-UPenn Partnership, Gaborone, Botswana; 5 Division of Infectious Diseases, Department of Medicine, Perelman School of Medicine, University of Pennsylvania, Philadelphia, Pennsylvania, United States of America; 6 Department of Clinical Research, Faculty of Infectious and Tropical Diseases, London School of Hygiene and Tropical Medicine, London, United Kingdom; 7 Division of Infectious Diseases and HIV Medicine, Department of Medicine, University of Cape Town, Cape Town, South Africa; 8 Institute of Infectious Disease and Molecular Medicine, University of Cape Town, Cape Town, South Africa; 9 Institute of Microbiology and Infection and the School of Biosciences, University of Birmingham, Birmingham, United Kingdom; 10 National Institute for Health Research (NIHR) Surgical Reconstruction and Microbiology Research Centre, University Hospitals of Birmingham National Health Service (NHS) Foundation Trust, Queen Elizabeth Hospital Birmingham, Birmingham, United Kingdom; University of California San Diego School of Medicine, UNITED STATES

## Abstract

Cryptococcal meningitis is a major cause of mortality throughout the developing world, yet little is known about the genetic markers underlying Cryptococcal virulence and patient outcome. We studied a cohort of 230 *Cryptococcus neoformans* (*Cn*) isolates from HIV-positive South African clinical trial patients with detailed clinical follow-up using multi-locus sequence typing and *in vitro* phenotypic virulence assays, correlating these data with clinical and fungal markers of disease in the patient. South African *Cn* displayed high levels of genetic diversity and locus variability compared to globally distributed types, and we identified 50 sequence types grouped within the main molecular types VNI, VNII and VNB, with 72% of isolates typed into one of seven 'high frequency' sequence types. Spatial analysis of patients’ cryptococcal genotype was not shown to be clustered geographically, which might argue against recent local acquisition and in favour of reactivation of latent infection. Through comparison of MLST genotyping data with clinical parameters, we found a relationship between genetic lineage and clinical outcome, with patients infected with the VNB lineage having significantly worse survival (n=8, HR 3.35, CI 1.51-7.20, p=0.003), and this was maintained even after adjustment for known prognostic indicators and treatment regimen. Comparison of fungal genotype with *in vitro* phenotype (phagocytosis, laccase activity and CSF survival) performed on a subset of 89 isolates revealed evidence of lineage-associated virulence phenotype, with the VNII lineage displaying increased laccase activity (p=0.001) and *ex vivo* CSF survival (p=0.0001). These findings show that *Cryptococcus neoformans* is a phenotypically heterogeneous pathogen, and that lineage plays an important role in cryptococcal virulence during human infection. Furthermore, a detailed understanding of the genetic diversity in Southern Africa will support further investigation into how genetic diversity is structured across African environments, allowing assessment of the risks different ecotypes pose to infection.

## Author Summary

*Cryptococcus neoformans* (*Cn*) is a yeast that commonly causes meningitis in HIV infected individuals in Africa, where it may account for up to 500,000 deaths every year. In this highly translational and multidisciplinary study, we used genetic analysis techniques to show that *Cryptococcus* found in Southern Africa represents a hotspot of genetic diversity. We combined this data with the results of microbiological techniques that assess the natural virulence traits that the yeast uses to survive and infect humans to further show that genetic diversity is associated with differences in cryptococcal phenotype. Finally, we analysed detailed clinical data on patients to investigate the clinical effects of infection with different lineages, and showed that one genetic lineage (VNB) is significantly associated with worse survival. Whilst much of our prior knowledge regarding the genetic basis of virulence is derived from studies on laboratory-adapted cryptococcal strains, our findings from this large and comprehensive MLST genotyping study of clinical isolates—linking genotype, phenotype, clinical presentation and outcome—provide direct insights into the contribution of pathogen lineage to virulence in human cryptococcal meningitis.

## Introduction

Cryptococcal meningitis (CM), caused by the fungus *Cryptococcus neoformans* (*Cn*) is one of the major causes of AIDS-related mortality, particularly in sub-Saharan Africa, where it was estimated to account for as many as 500,000 deaths annually prior to wide scale availability of antiretroviral therapy (ART) [1]. Despite widening access to ART, acute mortality from HIV-associated CM in Sub-Saharan Africa ranges between 20 and 50% within the optimised setting of clinical trials [2], reaching as high as 70% in real-world practice [3,4]. A number of clinical adverse prognostic markers in HIV-associated CM have been identified, including high fungal burden at CM diagnosis, poor rate of cryptococcal clearance from patient cerebrospinal fluid (CSF) during antifungal treatment, and altered mental status at presentation [5,6].

Although these clinical parameters are associated with poor outcome, the mechanisms underpinning this variation have not yet been fully explored. Patient-to-patient differences in clinical phenotype likely reflect a complex interplay between host factors (level of immunosuppression, immune response phenotype [6], underlying immunogenetics [7]) and pathogen virulence factors, as well as health system factors such as delays to diagnosis and treatment. We recently demonstrated an association between the *in vitro* phagocytosis of clinical cryptococcal isolates by J774 murine macrophage-like cells and poor patient survival in a cohort of 65 patients with HIV-associated CM [8]. These phenotypic differences are likely the result of genetic variation between isolates. Since *Cryptococcus* sp. are environmental, and infection is not horizontally transmitted between patients (infections are opportunistic and the human is a dead-end host), it is likely that virulence traits enabling *Cn* infection to occur are a result of the organism’s innate ability to survive in the human, perhaps developed as a defence against environmental predation [9]. Consequentially, long term natural selection of *Cn* by human antimicrobial defences does not occur, with the attendant likelihood that virulence factors will demonstrate natural variation within and amongst lineages. Previous studies in other pathogenic microorganisms and fungi have shown evidence of genetic lineage associated with phenotype and clinical outcome [10–12], and a recent study of 140 *Cn* isolates from Uganda provided the first evidence of this in *Cryptococcus* [13].

Of the two cryptococcal species known to infect humans (*C*. *gattii* and *Cn*), the majority of the global disease burden is caused by the *Cn* var *grubii* (*Cng*) subspecies infecting severely immunocompromised HIV-infected individuals [14]. *Cng* can be broadly divided into three major molecular types: the first two, VNI and VNII, are globally distributed with a third, VNB, appearing to be endemic to Southern Africa [14,15], although there is growing evidence of globally distributed VNB [16,17]. There are a number of genotyping schemes available for *Cng*, but in 2009 the International Society for Human and Animal Mycology (ISHAM) Working Group agreed upon a standardised approach using multi locus sequence typing (MLST) of seven 400–700 bp loci (with a discretionary eighth locus; TEF1) believed to be under neutral selective pressure [18]. MLST has been extensively used to analyse the evolutionary relatedness between isolates and studies have shown that disproportionate levels of genetic diversity, both at the level of the nucleotide and lineage, are present in cryptococcal isolates isolated from southern Africa when compared to isolates from around the globe [19,20]. Such discrepancies in the regional diversity of *Cn*, along with the discovery of sequences that could be considered ‘ancestral’ in southern Africa, have led to the proposal of an ‘out of Africa’ hypothesis, in which a small number of genotypes from within the broader diversity of African populations have been spread globally, leading to a clonal expansion of *Cn* worldwide [19–21].

In this study, we genotyped 230 *Cng* isolates from well-characterised patients enrolled in four clinical trials conducted in South Africa using MLST, and studied the genetic diversity of *Cn* present in the patients from the region. In a subset of 89 patients, we explored associations between genotype and *in vitro* phenotype. Finally, we analysed the whole patient cohort and looked for relationships between genotype, clinical presentation and outcome of CM. We hypothesised that genotype was associated with cryptococcal virulence, and that if true, we would find a correlation between phenotypic and clinical parameters, and the underlying lineage and genotype of *Cn*.

## Methods

### Patients/Isolates

Clinical Cn isolates were obtained as part of four clinical trials conducted in South Africa (Cape Town and Pietermaritzburg, Kwazulu-Natal) between 2005–2010 [22–25]. *Cn* was isolated from the CSF of HIV infected individuals prior to the initiation of antifungal treatment by plating CSF onto Sabouraud Dextrose (SD) agar (Oxoid, Fisher Scientific) and growing at 30°C for 48 hours. A representative sample of the cryptococcal population was stored by taking a broad ‘sweep’ of all colonies on the plate and stored in cryopreservative medium (80% SD broth, 20% glycerol) at -80°C until required for further testing.

### Molecular Testing

Glycerol stocks of Cn isolates were plated onto SD agar and grown at 30°C for 72 hours. Single colonies were selected and inoculated in 5 ml SD liquid media, followed by incubation at 37°C with agitation (165 rpm) for 16 hours. Fungal DNA was extracted using the MasterPure Yeast DNA Purification kit (Epicentre) according to the manufacturer’s instructions, but with the addition of two cycles of rapid bead beating (45 secs, 4.5 m/sec) using a Ribolyser Homogenizer (Hybaid, Middlesex UK) prior to the heat inactivation step. Genomic DNA was amplified by PCR using MLST primers for seven loci (*CAP59*, *GPD1*, IGS1, *LAC1*, *PLB1*, *SOD1*, *URA5*) according to the method of Meyer and colleagues [18]. PCR products were purified using a standard polyethylene-glycol/NaCl method and sequenced on an ABI3730xl DNA Analyser (Life Technologies) using Big Dye v1.1 chemistry at MRC Clinical Genomics Centre (Hammersmith, London, UK). Mating type was determined using by PCR according to the method of Barretto de Oliveira and colleagues [26].

### Phenotyping

Genotyping had not been completed when previous phenotyping work was performed [8], and therefore samples were not evenly selected or distributed across genotypes. For the current analysis, sufficient samples to represent each major MLST type were randomly selected and phenotyped to ensure a minimum of 5 samples of each genotype. *In vitro* phenotyping was performed on a further 32 samples using the CSF survival, laccase activity, and phagocytosis (macrophage uptake) assays as described in detail previously [8]. Briefly, CSF survival was tested using a standardised *Cn* inoculum incubated in pooled human CSF at 37°C for 96 hours, with sampling, plating and CFU counts performed every 12 hours, with the results plotted as a survival slope. Laccase activity was measured by inoculation and incubation of *Cn* in L-DOPA containing culture medium to induce melanin production. Pigment secreted into culture medium was measured by spectrophotometry, and all results were normalised to that of the H99 reference strain and expressed as a ratio. Phagocytosis was measured *in vitro* by infecting J774 macrophage-like murine cells (obtained from the European Collection of Cell Cultures) in 24-well culture plates with a multiplicity of infection of 10 *Cn* cells opsonised with monoclonal anti-capsule (18B7). After 2 hours, macrophages were washed, and internalised *Cn* cells released by lysing the macrophages with water and counted using a Haemocytometer. Two to six replicates were performed for laccase and phagocytosis assays, and all phenotypic data was analysed using the median of all replicates.

### Data Analysis

DNA sequences were assembled using CodonCode Aligner (CodonCode Corporation) and consensus sequences aligned using BioEdit software [27]. MLST alleles and sequence types were assigned by comparison with reference alleles using BLAST software [28]. Novel alleles were submitted to mlst.mycologylab.net for assignment of allele and sequence types. Examples of each MLST locus were submitted to the European Nucleotide Archive (www.ebi.ac.uk; Accession numbers LN812026-LN812094). Phylogenetic analyses were performed on concatenated MLST loci sequences using RAxML (GTR gamma substitution model, sequence partitioned by loci, 1000 bootstraps performed using rapid hill-climbing methodology; [29]). For the purposes of molecular analysis, the IGS1 sequence was removed from all concatamers over the aligned region of indels (nt 1306–1829 in H99) in order to avoid excessive bias due to insertions/deletions (indels). Major molecular types were assigned according to phylogenetic clade in conjunction with agreed reference type strains available online (H99 and WM148, VNI; WM626, VNII; Bt1, VNB). BURST analysis was performed using eBURST [30] with branches defined based on up to two allele changes. SplitsTree4 [31] was used to generate a neighbour network from the nucleotide sequence alignment. Analysis in STRUCTURE(v2.3.4) was performed using the admixture model, allowing alpha to be inferred and assuming correlated allele frequencies, with a Burnin period of 10,000 Markov chain Monte Carlo (MCMC) replications followed by 10,000 sampling replications [32]. Twenty runs of STRUCTURE analyses were performed for K values 1–20, and data were analysed using the method of Evanno and colleagues as implemented in StructureHARVESTER [33,34]. The same conditions in STRUCTURE were applied to the linkage model, and the results were compared with those of the admixture model and found to be equivalent. A consensus STRUCTURE plot was obtained from the admixture repeats using the greedy algorithm in CLUMPP [35], and final plots were produced in STRUCTURE PLOT [36].

Address information was available for most patients, but due to the temporary nature of some township dwellings, as well as ongoing urban redevelopment, this information was incomplete. Google Maps and the City of Cape Town interactive viewer (www.capetown.gov.za) were used to determine GPS coordinates based on full address or property identifier linked to ward. In a very small proportion of cases, a specific location could not be determined, and the GPS coordinate was mapped to the centre of the nearest matching ward or district. Spatial data was anonymised and converted to a distance matrix for analysis (S2 File). The possible presence of spatial autocorrelation between concatenated MLST sequences was tested using a Mantel correlogram using R package ‘vegan’ (Version 2.2.1) [37] and a genetic variogram using R package ‘phylin’ (Version 1.0)[38] in R (Version 3.1.2)[39]. See S1 File for more details.

Statistical analyses of clinical and molecular data were performed using STATA 13.1 (StataCorp. 2007. *Stata Statistical Software*. College Station, TX: StataCorp LP.). Most phenotypic and clinical analyses were performed on non-parametric data. Groups were compared using Kruskal-Wallis test for continuous data and Chi square test for Boolean data. Comparison of continuous variables to one another (such as phenotypic variables) was performed using linear regression. Patient survival data was tested using Cox’s proportional hazard analysis, adjusting for known adverse prognostic indicators. Multivariate linear regression models were constructed by initially determining clinical and microbiological factors associated in univariate analysis (P<0.1), and then added to the model in stepwise fashion as previously described [6].

### Ethics Statement

Ethical approval was obtained from the research ethics committees of The Faculty of Health Sciences, University of Cape Town, The Medicines Control Council of South Africa, The University of Kwazulu-Natal, Edendale Hospital, the Kwazulu-Natal Department of Health, and by Wandsworth local research ethics committee, covering St George’s Hospital, UK. Informed written patient consent was obtained for further testing of isolates as part of the clinical trial protocol.

### Accession Numbers

EMBL Accession Numbers for sequences: LN812026, LN812027, LN812028, LN812029, LN812030, LN812031, LN812032, LN812033, LN812034, LN812035, LN812036, LN812037, LN812038, LN812039, LN812040, LN812041, LN812042, LN812043, LN812044, LN812045, LN812046, LN812047, LN812048, LN812049, LN812050, LN812051, LN812052, LN812053, LN812054, LN812055, LN812056, LN812057, LN812058, LN812059, LN812060, LN812061, LN812062, LN812063, LN812064, LN812065, LN812066, LN812067, LN812068, LN812069, LN812070, LN812071, LN812072, LN812073, LN812074, LN812075, LN812076, LN812077, LN812078, LN812079, LN812080, LN812081, LN812082, LN812083, LN812084, LN812085, LN812086, LN812087, LN812088, LN812089, LN812090, LN812091, LN812092, LN812093, LN812094.

## Results

### Allele Types, MLST Types, Molecular Types and Diversity/Distribution

**Fig 1:**
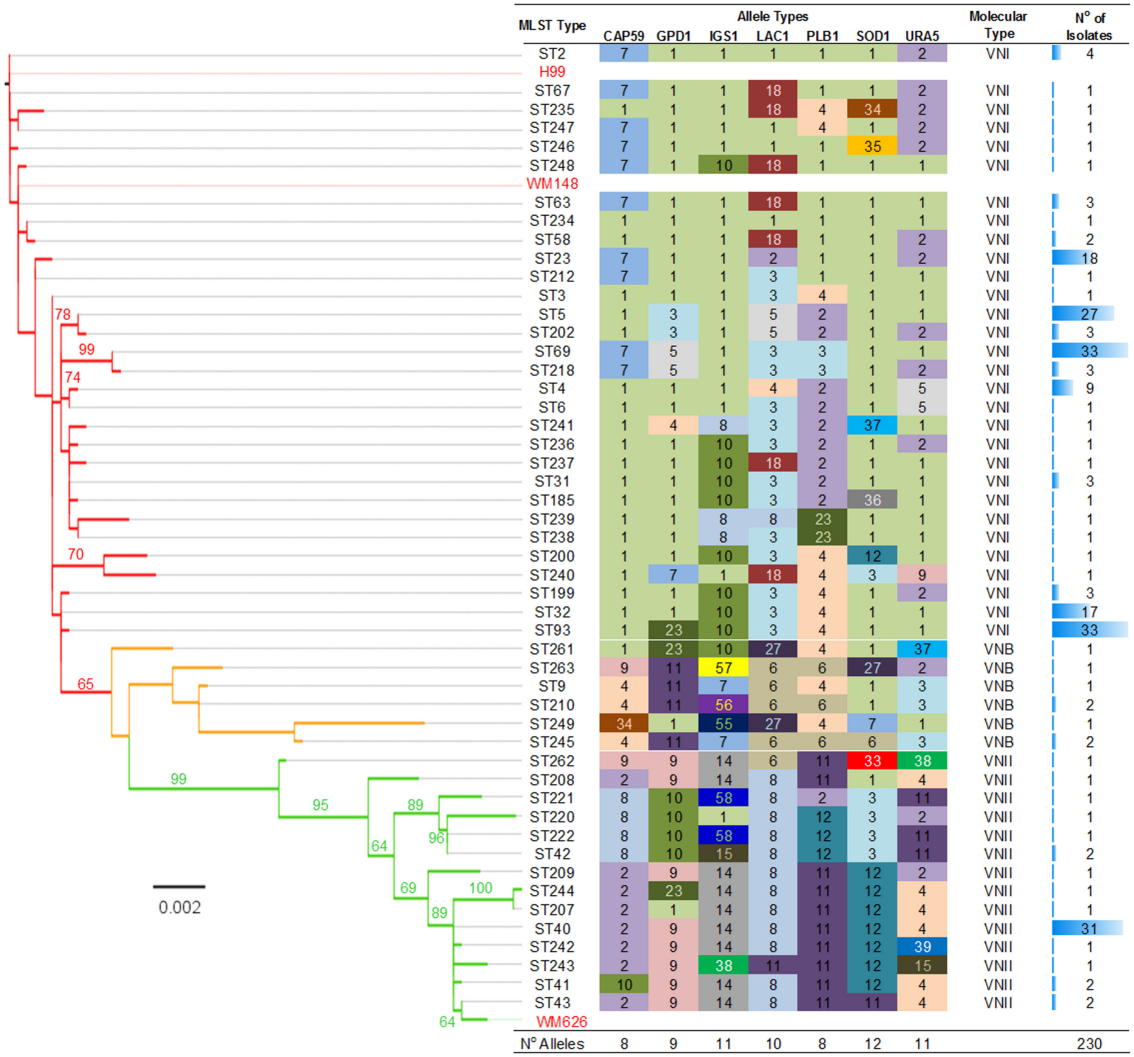
Assignment of MLST alleles, phylogeny and distribution of clinical isolates. Unrooted, ordered maximum likelihood tree (RAxML, GTR gamma, partitioned by loci) using concatenated nucleotide sequences from 7 loci and one representative sequence for each MLST type. Bootstrap values are shown for branches with greater than 60% agreement between replicates (1000 replicates). Also showing allele typing and MLST assignment for each sequence type, molecular type assignment according to phylogeny, and number of isolates belonging to each ST type.

Single colonies of *Cryptococcus neoformans* isolated from CSF of 230 HIV-positive clinical patients were sequenced for all seven loci according to the ISHAM typing scheme [18], and MLST genotypes assigned (Fig 1). MLST profiling of *Cn* revealed great genetic diversity within South African patients, with 50 different sequence types (STs), including at least 18 novel STs. One hundred and sixty eight (72%) of isolates were typed as one of seven ‘high frequency’ genotypes containing 9 or more examples (ST4, 4%; ST5, 12%; ST23, 8%; ST32, 7%; ST69, 14%; ST93, 14%; ST40, 13%), with the remaining samples (n = 62) broadly distributed into 43 ‘low frequency’ genotypes (Fig 1). Mating type was successfully obtained for 229 isolates, of which 226 were found to be type *MAT*α, with the remaining 3 isolates (each of different ST: ST238, ST239, ST249; two VNI, one VNB) having the *MAT*a type.

A small number of extremely unusual IGS1 sequences were identified: IGS1 allele types 55, 56, 57 and 58 were all closely related sequences that demonstrated large indels in relation to all other known IGS1 sequences, and large parts of the IGS1 allele sequence could not be effectively aligned with the other reference alleles. Analysis of the allele types reported at mlst.mycologylab.net showed that types 56 and 58 have only been described in ST210, ST221 and ST222, whilst allele types 55 and 57 (reported here in ST249 and ST263) have also previously been described in ST225 and ST224. The degree of change affecting a single locus meant that whilst allelic analyses based on MLST were unaffected, phylogenetic analyses based on nucleotide variation were heavily biased to classify isolates that contained these alleles as outliers. To overcome this, the affected region of IGS1 was removed from the concatamer of all sequence types (ST) prior to phylogenetic analysis.

Concatenated nucleotide sequences for all loci were used to generate an unrooted maximum likelihood tree (Fig 1), allowing broad phylogenetic clades (molecular types) to be determined that included all MLST STs. *Cn* Isolates were then categorised by molecular type, with the largest proportion being VNI (n = 175, 76%), followed by VNII (n = 47, 20%) and VNB (n = 8, 4%) (Fig 1). Of the 50 sequence types identified, 30 were found to be VNI, with most VNI samples (n = 128, 73%) grouping into one of the high frequency sequence types (ST5, ST23, ST32, ST69 and ST93) described earlier. The VNII molecular type contained 14 sequence types, the majority of which (n = 31, 66%) were of the high frequency ST40. The relatively low frequency VNB molecular type did not contain any high frequency sequence types, with eight isolates divided amongst six sequence types.

**Fig 2:**
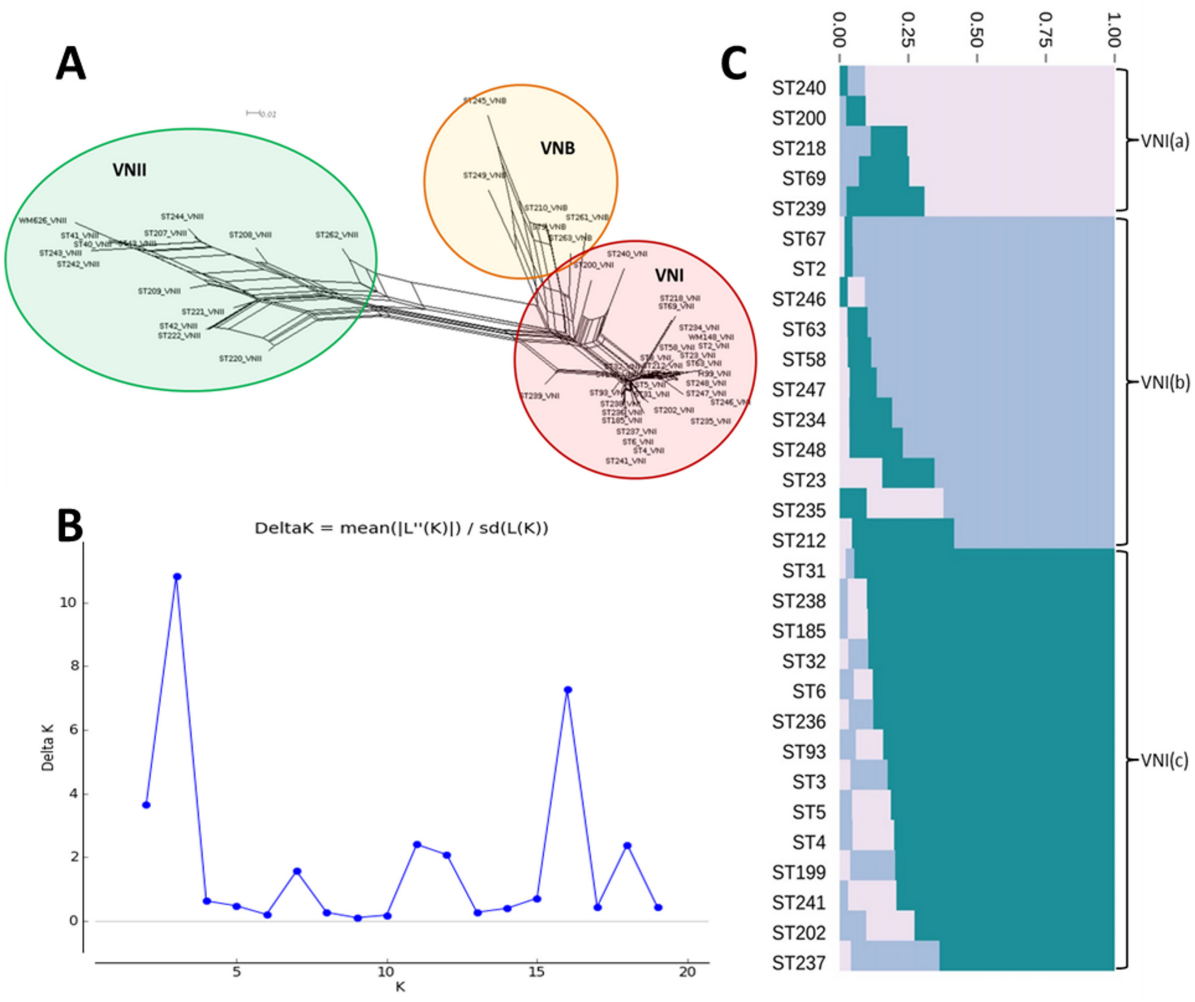
Phylogenetic and Bayesian analysis of concatenated nucleotide sequences. (A) SplitsTree neighbour network shows diverse VNII and VNB, and clonal VNI clades. (B) Analysis of VNI clustering using StructureHARVESTER and ΔK shows optimal number of K clusters is 3. (C) Use of STRUCTURE allows VNI sequences to be subdivided into 3 distinct populations: VNI(a), VNI(b), VNI(c).

Since our dataset contained isolates belonging to 50 different MLST types, many of which contained only one or two isolates (thus limiting statistical power for detecting associations), it was necessary to determine a rationale for grouping samples. We chose to analyse isolates grouped by molecular type and by the high frequency MLST types (Fig 1). Previous authors have used BURST grouping to cluster isolates [13,21,40], and although we considered applying the same methodology, eBURST grouping [30] of our dataset identified at least 10 distinct and unrelated clusters, most dominated by a single high frequency MLST type along with a very small number of ancillary types, and such groupings did not add power to the analyses over using individual sequence types (Fig A in S1 File). We instead used SplitsTree [31] to define molecular types, and found the VNI clade to be highly clonal (Fig 2A). To further study the population distribution of the VNI clade, we analysed the nucleotide sequences with an admixture model in STRUCTURE [32], and evaluated the optimal number of clusters using StructureHARVESTER [33,34]. This showed the optimal number of clusters (k) within VNI to be three (Fig 2B), and demonstrated distinct subpopulations in our VNI dataset, here defined as VNI(a), VNI(b) and VNI(c) (Fig 2C).

### Spatial Analysis of Clinical *Cn* Isolates

Out of 230 South African clinical isolates, 185 were from patients resident in Cape Town in the Western Cape Province. From those patients, 46 different sequence types were found within the population of a single city. The remaining 45 isolates were from patients in the KwaZulu-Natal province, where 13 sequence types were found, the most common of which was ST93 (n = 22, 49%), with four sequence types not present in patients from Cape Town (ST236, ST238, ST241, ST242). The VNB molecular type was only found in clinical isolates from Cape Town, and was not found in Kwazulu-Natal.

*Cryptococcus* is thought to be acquired from the environment, and it is likely that human exposure is nearly universal [41]. However, it remains unclear whether clinical CM is caused by recent infection or reactivation and dissemination of previously latent *Cn*. To explore the possibility of geographical clustering, indicating a possible source of local acquisition, patient addresses in Cape Town were mapped using GPS coordinates, and the concatenated MLST sequences were tested for spatial autocorrelation using a Mantel correlogram [42] and a genetic variogram (Fig B in S1 File)[43]. No significant spatial autocorrelation could be found at α = 0.05 and semivariogram modelling produced variogram models with a very short range and poor fit to the data (Fig C in S1 File), indicating an absence of spatial correlation between genetic and geographic distance in this study.

### Phenotypic Associations

In previous studies we explored a number of fungal virulence factors that can be characterised in the laboratory. These include the activity of the fungal enzyme laccase that catalyses the formation of melanin, the ability of *Cryptococcus* to survive in the hostile and resource-limited environment of *ex vivo* human CSF, and the tendency to be phagocytosed by macrophages. We reported how isolates that are highly phagocytosed by macrophage-like cells *in vitro* are counter-intuitively associated with poor patient survival and increased baseline fungal burden *in vivo* [8], and how *in vitro* phagocytosis is further associated with enhanced *in vitro* laccase activity and enhanced survival in *ex vivo* CSF. We hypothesised that isolates displaying phenotypic traits of being highly phagocytosed, with good CSF survival and high laccase activity *in vitro*, represent a high virulence phenotype associated with worse outcome in human CM. Fifty-seven *Cn* isolates from this previous study were included in our genotyped South African cohort. However, samples were not sufficiently distributed amongst genotypes to allow us to probe the relationships between genotype and *in vitro* phenotype. Therefore, a further 32 isolates were selected from within the cohort based solely on MLST type, and phenotyped using laccase activity, CSF survival and phagocytosis assays described previously [8]. The resulting set of 89 phenotyped isolates included a minimum of 5 isolates in each molecular type and high frequency MLST type, along with a small number of samples from low frequency MLST types (Table 1).

**Table 1:** *In vitro* phenotyping results ordered by molecular type, VNI subtype, and high frequency MLST type.

Molecular type	VNI Subtype	MLST type (high frequency)	N° Isolates	Laccase Activity (normalised to H99N)		CSF Survival (Slope CFU/ml /day)		Macrophage Uptake (cells/μl)	
				median	range	median	range	median	range
**VNI**			67	0.34	(0.02–1.12)	-1.33	(-3.07–-0.28)	313	(80–1278)
	**VNI(a)**		13	0.27	(0.02–1.12)	-0.79	(-1.95–-0.28)	270	(85–538)
		**ST69**	11	0.25	(0.02–1.12)	-0.79	(-1.77–-0.28)	250	(85–538)
	**VNI(b)**		12	0.14	(0.03–0.83)	-1.8	(-2.34–-0.88)	517	(165–771)
		**ST23**	5	0.12	(0.03–0.44)	-1.85	(-2.34–-1.14)	530	(165–705)
	**VNI(c)**		42	0.35	(0.05–0.91)	-1.28	(-3.07–-0.41)	312	(80–1278)
		**ST4**	9	0.52	(0.11–0.88)	-0.86	(-2.23–-0.52	465	(86–1278)
		**ST5**	10	0.41	(0.06–0.91)	-1.05	(-2.22–-0.53)	383	(92–614)
		**ST32**	10	0.41	(0.13–0.54)	-1.38	(-2.82–-0.91)	199	(112–605)
		**ST93**	10	0.27	(0.05–0.63)	-1.92	(-3.07–-0.96)	297	(80–515)
**VNII**			14	0.66	(0.32–1.06)	-0.38	(-1.01–-0.06)	285	(88–420)
		**ST40**	10	0.71	(0.32–1.06)	-0.22	(-0.91–-0.06)	266	(135–350)
**VNB**			8 (7)*	0.16	(0.03–0.91)	-1.44	(-2.32–-0.96)	250	(160–400)
**All**			89 (88)*	0.36	(0.02–1.12)	-1.11	(-3.07–-0.06)	300	(80–1278)

* CSF survival was not performed for one of the eight VNB isolates.

**Fig 3:**
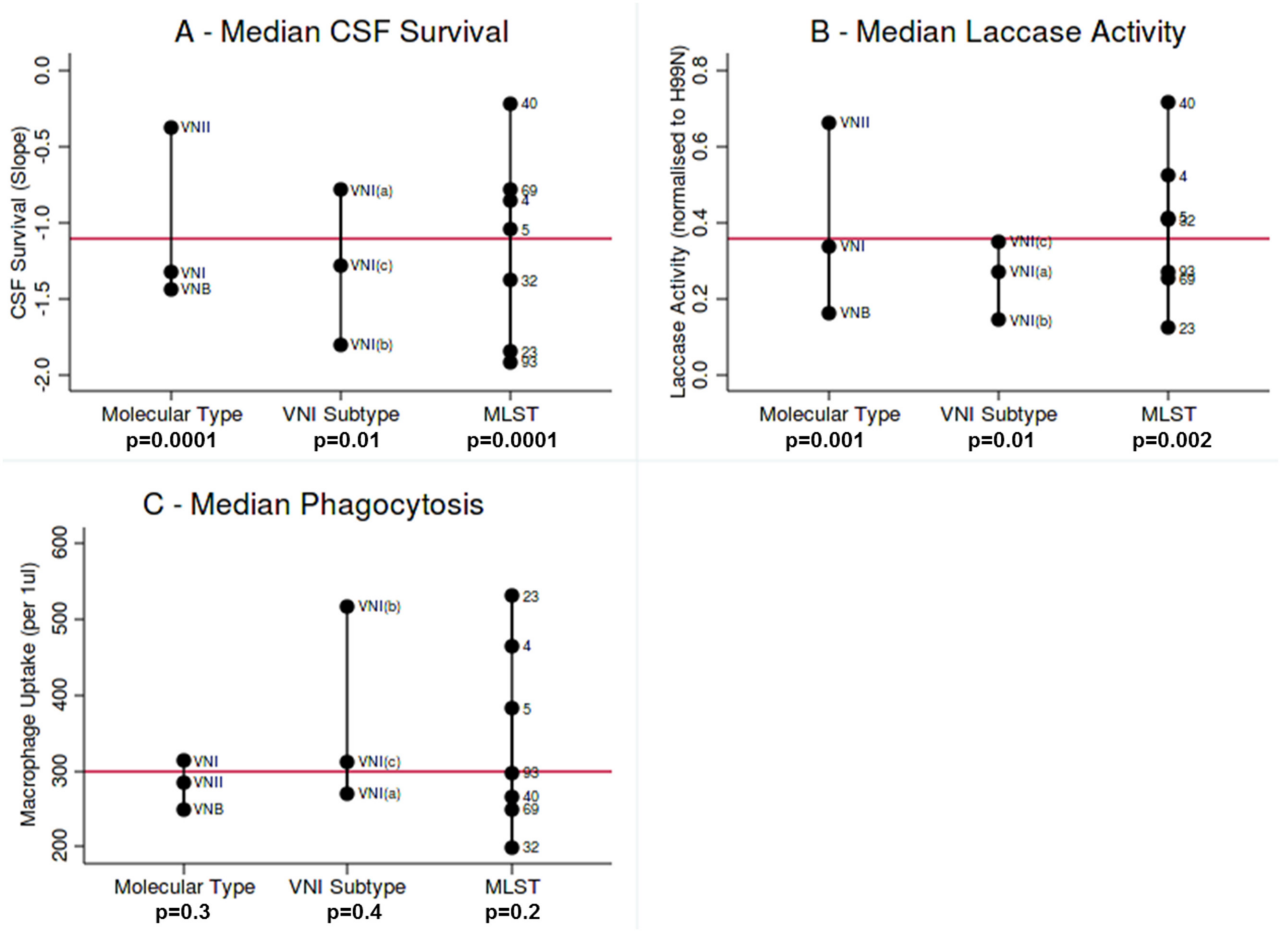
Median *in vitro* phenotyping values ordered by molecular type, VNI Subtype and high frequency MLST. Kruskal Wallis analysis performed on groups, and p values shown. Overall median is plotted in red. (A) Survival in *ex vivo* CSF, (B) Laccase Activity normalised to H99 reference strain, (C) *In vitro* phagocytosis of isolates by J774 cells (per 1 μl lysate).

We then compared the phenotypic data with genotype to test for lineage-associated phenotypes. When analysed by molecular type, *in vitro* survival in *ex vivo* human CSF differed significantly (Fig 3A, p = 0.0001), with the VNII group having a more shallow survival slope, indicating significantly better CSF survival (median CSF survival slope -0.38 log_10_ CFU/ml /day) compared to VNI (-1.33) and VNB (-1.11). Laccase production also differed significantly between groups (Fig 3, p = 0.001), with results normalised to the lab-adapted reference strain H99 indicating that VNII isolates had higher laccase activity (0.66 ratio to H99) compared to VNI (0.34) and VNB (0.16). These two phenotypes were correlated in our previous study [8], and remained so in this expanded dataset (p = 0.0001). There were no significant differences in *in vitro* phagocytosis by macrophages by molecular type.

Analysis within VNI molecular type by subtypes VNI(a), VNI(b) and VNI(c), indicated significant differences in CSF survival (Fig 3A, p = 0.01) but not laccase activity or *in vitro* phagocytosis. Analysis by high frequency MLST type also indicated phenotypic variability that was significant for CSF survival (Fig 3A, p = 0.0001) and laccase activity (Fig 3B, p = 0.002), and even when the outlying VNII ST40 group was removed, differences in CSF survival between VNI STs were still present (p = 0.03). Although the VNII molecular type and the VNII ST40 appeared to exhibit both high laccase activity and good CSF survival, no particular MLST type consistently exhibited a ‘high virulence’ triad of being highly phagocytosed, with high laccase activity and good CSF survival.

### Clinical Associations

We next sought to determine whether clinical parameters and outcome in human CM were associated with genetic lineage. Of the 230 patients who had their clinical *Cn* isolates genotyped in this study, 226 patients received amphotericin B-based induction antifungal therapy, with just four patients receiving fluconazole without amphotericin B. Since fluconazole treatment alone is known to be associated with higher mortality [6], we removed these four patients from the survival analyses. Where available, details of baseline patient characteristics, CSF findings and mortality are shown in supplementary information across the whole cohort and split by major molecular groupings (S1 Table), and by isolate (S3 File).

**Fig 4:**
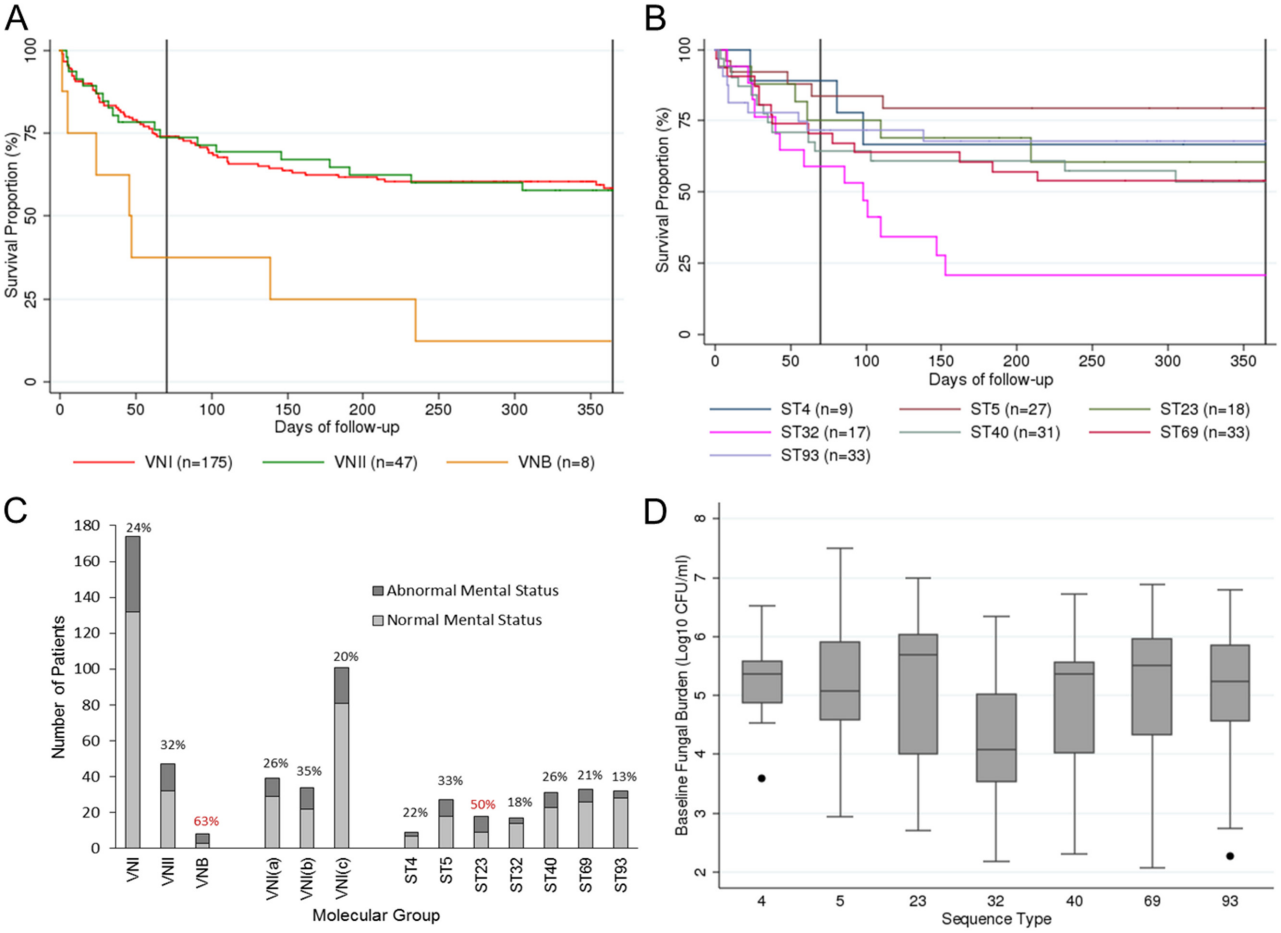
Clinical characteristics and survival of patients infected with different lineages of *C neoformans*. (A) Patient survival by molecular type; VNB isolates (n = 8) were associated with significantly worse outcome compared to VNI and VNII isolates at 70 days (p = 0.01) and 365 days (p = 0.003) follow-up in Cox’s proportional hazard survival analysis. (B) Patient survival by high frequency MLST type. (C) Number of patients with and without altered mental status by molecular group (% with altered mental status indicated above each bar). (D) Baseline fungal burden, showing variation by high frequency MLST type.

Long-term survival outcome data was available for all patients, with an overall mortality of 27% at 10 weeks, and 41% at one year. We explored the relationship between patient outcome and cryptococcal genotype. When samples were analysed by molecular type, survival was similar in patients infected with isolates belonging to the predominant VNI and VNII molecular type (Fig 4A), whilst the 8 patients infected with *Cn* isolates of the VNB molecular type had significantly higher mortality at 10 weeks (HR 3.20, CI 1.26–8.09, p = 0.014) and at one year (HR 3.35, CI 1.51–7.20, p = 0.003). This difference was maintained even following adjustment for previously published adverse prognostic factors [6] of age, weight, baseline haemoglobin, CD4 count, baseline fungal burden and altered mental status, as well as differences in treatment regimen (duration of amphotericin B induction therapy, treatment with fluconazole, flucytosine, or voriconazole as a second drug, adjunctive IFN-γ) in a multivariate analysis (10 week aHR 4.02, CI 1.28–12.67, p = 0.018; 1 year aHR 3.92, CI 1.53–1.006, p = 0.005). Single variable and multivariate analysis within VNI molecular type showed no significant survival differences between the VNI(a), VNI(b) and VNI(c) subtypes.

Given that the VNB molecular type is a rare cause of CM globally, we then explored survival in patients infected with the high frequency South African MLST types found within the globally distributed VNI and VNII molecular types. There was limited evidence for survival differences between patients infected with different cryptococcal MLST types (Fig 4B), with patients infected with ST32 showing worse survival (p = 0.077) after one year in bivariate but not multivariate analysis.

To examine these differences in survival by molecular or sequence type, we compared clinical parameters known to be important determinants of outcome in human CM across genotypes. Patients infected with VNB type were more likely to have altered mental status (Fig 4C; p = 0.04). There were no significant differences in baseline fungal burden, rate of clearance of infection or CSF white cell count between patients infected with different molecular types or VNI subtypes.

Clinical parameters varied by high frequency MLST type (Fig 4C and 4D). The apparent association between infection with ST32 and poor survival could not be explained by adverse patient clinical parameters, and ST32 isolates had amongst the lowest baseline fungal burdens of all groups (Fig 4D). Nor did phenotyping of ST32 reveal attributes of high phagocytosis, high laccase activity or good CSF survival. Conversely, ST40 isolates within VNII, which based on *in vitro* phenotyping might have been expected to be more virulent, showed little difference in clinical parameters or long-term survival (Fig 4B, 4C and 4D).

In other high frequency sequence types, variations were apparent in baseline fungal burden, and rate of clearance was significantly different between ST types (p = 0.0001; S1 Table). On bivariate regression, ST5 was associated with faster rate of clearance from CSF (p = 0.04); however this association was not maintained following adjustment for other factors associated with rate of clearance (baseline fungal burden, treatment with amphotericin B, addition of flucytosine, fluconazole or IFN-γ) on multivariate analysis.

## Discussion

MLST characterisation of a large number of *Cryptococcus neoformans* isolates causing meningitis in HIV-infected South African patients revealed high genetic diversity in this region, with fifty discrete STs in 230 patients. We found 46 different sequence types within patients living in Cape Town alone. This level of diversity was surprising, as another recent study genotyping 170 South African *Cn* isolates found just 23 different sequence types, although this study did not use the ISHAM typing scheme [15]. These South African studies represent a marked contrast with most other MLST studies, which have typically reported a relatively low number of sequence types present in each geographical location [13,21,40,44]. For example, a large recent study of over 476 clinical and environmental isolates obtained from multiple sites in 8 countries across Asia and the Middle East found just 28 sequence types, with most countries dominated by 1–3 high frequency genetic lineages [40]. Furthermore, the diversity we observed was not confined to changes in a single locus, but represented changes in multiple loci, such that BURST grouping was not a feasible approach for analysis. Despite this diversity, 73% of the isolates (n = 168) were genotyped as one of seven ‘high frequency’ sequence types, with the remaining 26% of isolates (n = 62) genotyped into 43 different sequence types with between one and four examples of each type.

To understand the possible sources of such broad diversity, we mapped patient address data using GPS coordinates. This revealed that there was no clustering of MLST or molecular types within Cape Town—within our dataset, the high frequency MLST STs and the different molecular types were spread across patients from all the major townships of Cape Town investigated, and there was no correlation between spatial and genetic distance. This could perhaps argue against local acquisition. However, it is difficult to speculate why Cape Town might have such high sequence diversity within such a small location without performing environmental sampling to establish whether the same MLST types are present in the local environment. One possible explanation for the observed diversity is that the populations of the Cape Town are historically transient and migratory, with a very high proportion of residents migrating to Cape Town from rural areas in the Eastern Cape during the early post-apartheid era [45]; unfortunately information regarding place of birth was not available for individual patients. Patients with HIV-associated cryptococcosis might be experiencing reactivation of latent cryptococcal infection acquired months or years ago in their village of birth, although some people of Xhosa ancestry residing in Cape Town do return for regular annual visits to rural locations in the Eastern Cape, where re-exposure may occur. We did not have information regarding duration of residence at an address to make any firm conclusions. Molecular epidemiological studies have previously shown that patients emigrating from Africa to Europe may experience cryptococcal infection due to strains of African origin many years later [46,47], and therefore latency is a plausible explanation. Although clearly residents may circulate within and between townships, even with travel to the Eastern Cape it is unlikely that African patients are more mobile than Asian patients [40], indicating that the environmental diversity of southern African *Cn* is demonstrably higher than that found elsewhere in the world.

Phylogenetic analysis showed that although most isolates were of the globally distributed VNI molecular type, there were also a substantial number of VNII isolates, as well as the much less common VNB molecular type, which in the literature appears to be most commonly found in southern Africa. Other authors have used BURST analysis for clustering MLST data [13,21,30,40]. Whilst this is a reasonable approach, it is most effective when analysing a network of closely related sequences connected by changes to a single allele. Furthermore, BURST analysis is limited by the simplistic allelic definitions of MLST as well as not necessarily capturing the effects of recombination, and thus provides less resolution of sequence relatedness than that of a phylogeny based on concatenated nucleotide sequences. *Cn*, in particular VNB, is known to be highly recombinogenic [14,20,21] so instead of BURST grouping, we used STRUCTURE [32] to delineate three genetic lineages within VNI isolates and used these groupings as part of our analyses of phenotypic and clinical association with genotype.

We have previously used *in vitro* phenotyping to demonstrate the highly variable virulence of clinical *Cryptococcus neoformans* isolates. In this study we were able to compare *in vitro* phenotyping data with MLST genotyping data, and showed that these differences are associated with genetic lineage. In particular, VNII isolates, which are genetically distinct from VNI and VNB, are also phenotypically distinct, with significantly different laccase activity and survival in *ex vivo* human CSF. Within the globally distributed VNI molecular type, a population genetics approach to broadly subdivide isolates into clusters using STRUCTURE increased the sample size, and showed that one cluster (VNI(a)) had significantly better *ex vivo* CSF survival compared to VNI(b) and (c) subtypes. Differences were also detected at the level of the MLST ST, and whilst ST40 (as the representative VNII ST) might be expected to show differences, even when ST40 was removed from the analysis we still found differences in CSF survival.

The South African cohort of *Cn* isolates, from extensively clinically characterised clinical trial patients with long-term follow-up, provided an excellent resource for study. In this study, as well as investigating phenotypic variation, we explored the influence of genetic lineage on clinical factors. Given that 98% of patients received amphotericin-based induction CM therapy, differences in treatment regimen were not considered a major contributor to the clinical and survival variations observed, and indeed we removed those patients not receiving amphotericin B from the survival analyses. Furthermore, apart from rate of clearance and patient survival, all clinical parameters studied were from baseline, prior to commencement of anti-fungal treatment, and were therefore considered to be independent of drug regimen.

We initially analysed isolates according to molecular type, and found that the apparently rare VNB molecular type was associated with poor patient survival, even at the clinically relevant time-point of 10 weeks. The presence of only eight VNB isolates within the dataset means that this result must be viewed with some scepticism, even though the association of VNB with patient survival was maintained following adjustment for treatment regimen and baseline variables likely to be impacting on survival, including altered mental status, an important independent adverse prognostic marker [5,6] that was over-represented in this group. The pathophysiology of altered mental status remains poorly understood [6] and it is uncertain to what extent it is driven by pathogen or host factors. It is therefore plausible that altered mental status is on the causal pathway between VNB and mortality. Nevertheless, the differences in long-term outcome remained significant even after adjustment for all known CM adverse prognostic markers and treatment regimen. We sought to explain this survival difference within other clinical parameters, as well by studying the *in vitro* phenotype for a number of known virulence factors, but were unable to find a plausible explanation, since VNB isolates were clinically and phenotypically very similar to VNI isolates. We were thus unable to explain the underlying link between infection with VNB strains and worse outcome. The factors that underpin the apparent high virulence of VNB need to be further investigated and the findings replicated in a larger cohort. To date, the VNB molecular type has been most commonly described in Southern Africa, and may be a rare and unusual genotype; this lineage may be significantly different from the globally distributed VNI and VNII genotypes (which showed comparable survival curves).

Since observations relating to VNB may not be broadly applicable to global cryptococcosis, we analysed the dataset using the seven high frequency MLST types, each of which contained between 9 and 33 isolates. Although Fig 4B shows clear differences in patient survival between MLST types, with survival in ST32-infected patients particularly poor, these differences were not statistically significant. However, this may simply reflect the small sample size for each ST.

The broad genetic diversity of *Cn* isolates infecting patients precluded the comparison of large groups of highly related sequences, limiting the statistical power to detect genotype-phenotype associations. Clustering VNI isolates using STRUCTURE, although useful from a population genetics standpoint, did not demonstrate strong clinical or survival differences.

Another important caveat of this work is that we sequenced single colonies from each patient’s *Cn* isolate. Mixed infections have been reported in up to 20% of patients with CM [48], and by sequencing only a single colony we may have missed the lineage responsible for clinical and phenotypic results.

When both clinical and phenotypic characteristics are compared according to genetic lineage, it becomes clear that rather than being a homogenous group, different lineages of *Cryptococcus* exhibit a wide range of differences both within and between lineages, and that many of these differences may be the result of inherited traits. This raises important questions about the use of historically cultured and passaged reference strains such as H99 for inferring the significance of genes and in ‘knock-down’ studies, as well as the use of small numbers of representative ‘type’ strains to describe genotypes. We would advocate larger samples sizes with increased sample diversity, as well as ensuring comparative analyses are performed on samples of similar genetic background.

As our study shows, significant clinical and phenotypic differences are detectable between genetic lineages. However unravelling the complex relationships between pathogen genetic diversity and disease presentation and outcome in an immunocompromised host is difficult. Although this study represents a large number of clinical trial patients, the diversity within the dataset meant that genotypic groups were small, reducing the power of the analyses. Furthermore, other variables, such as host immunogenetics and immune response, delays to clinical presentation, variable access and adherence to antifungal and antiretroviral treatment, and the possibility of further opportunistic infections are all elements that impact on long-term survival and cannot be controlled for in a human population, particularly in the context of a developing world health-care system. Further work in an animal model would allow more controlled exploration of the influence of genetic diversity on clinical outcome, albeit with the caveats that such non-human models represent. Finally, MLST captures only a small fraction of the sequence diversity and poorly accounts for recombination. Whole genome sequencing is becoming increasingly affordable [49], and is currently underway for isolates from well-characterised patient cohorts. In sufficient numbers this will allow genome wide association studies to be performed, allowing identification of the alleles associated with pathogenesis and virulence, with the eventual aim of identifying novel drug targets.

## Supporting Information

S1 Table Characteristics of patients in the whole dataset, and by major molecular grouping. Samples grouped by molecular type, VNI subtype and high frequency MLST type. Values shown are median (range) for continuous variables, and number(percentage) for categoric variables and mortality. P values shown are Kruskal-Wallis scores for continuous data, Chi Square for Boolean data, and Cox’s Proportional Hazard Analysis for survival (for group with lowest p value).
(XLSX)

S1 File BURST and spatial analyses of isolates. Fig A in S1 File. eBURST analysis of South African VNI isolates within dataset identified 6 unrelated clusters. Fig B in S1 File Absence of strong spatial correlation with genetic distance. Fig C in S1 File Genetic variogram of South African samples.
(DOCX)

S2 File Spatial distance between isolates. Anonymised Spatial Distance matrix representation of patient address data between isolates (along top row and far left column) used in analysis, in tab-delimited format.
(TXT)

S3 File Detailed genotypic, clinical and phenotypic data by isolate. Data is ordered by columns in tab-delimited format.
(TXT)
