# Supplementary material for: Genotypic Diversity Is Associated with Clinical Outcome and Phenotype in Cryptococcal Meningitis across Southern Africa
Source: PLoS Negl Trop Dis. 2015 Jun 25;9(6):e0003847. doi: 10.1371/journal.pntd.0003847 (PMC4482434; doi:10.1371/journal.pntd.0003847)
Supplement: S1 File — Fig A in S1 File. eBURST analysis of South African VNI isolates within dataset identified 6 unrelated clusters. Fig B in S1 File Absence of strong spatial correlation with genetic distance. Fig C in S1 File Genetic variogram of South African samples. (DOCX) [file pntd.0003847.s002.docx]

# Supplementary Information S1 File

## BURST Analysis

BURST is an algorithm commonly used with MLST data for identifying mutually exclusive clusters of samples based on haplotype or locus identity (E.J. Feil, B.C. Li, D.M. Aanensen, et al, J. Bacteriol. **186**:1518-1530, 2004). It uses the allelic definitions of types of MLST (as shown in Fig 1), and groups samples according to differences between one or two loci, and is commonly used in a radial pattern to determine the ancestral type in a population. We used the eBURST3 program available at eburst.mlst.net to analyse the VNI allele types in Fig 1. eBURST analysis showed six clusters in VNI, five of which were dominated by the high frequency MLST types described in the primary text (ST4, ST5, ST23,ST32, ST69, ST93) (Fig A in S1 file). The remaining cluster contained two “low frequency” STs (cluster 4, ST238 and ST239). One cluster contained two high frequency STs (cluster 1, ST32 and ST93). With these exceptions, clusters were essentially defined by the high frequency MLST type found in them, with only a very small number of ancillary samples. Therefore, there was no benefit to adding these ancillary sequences to analyses.


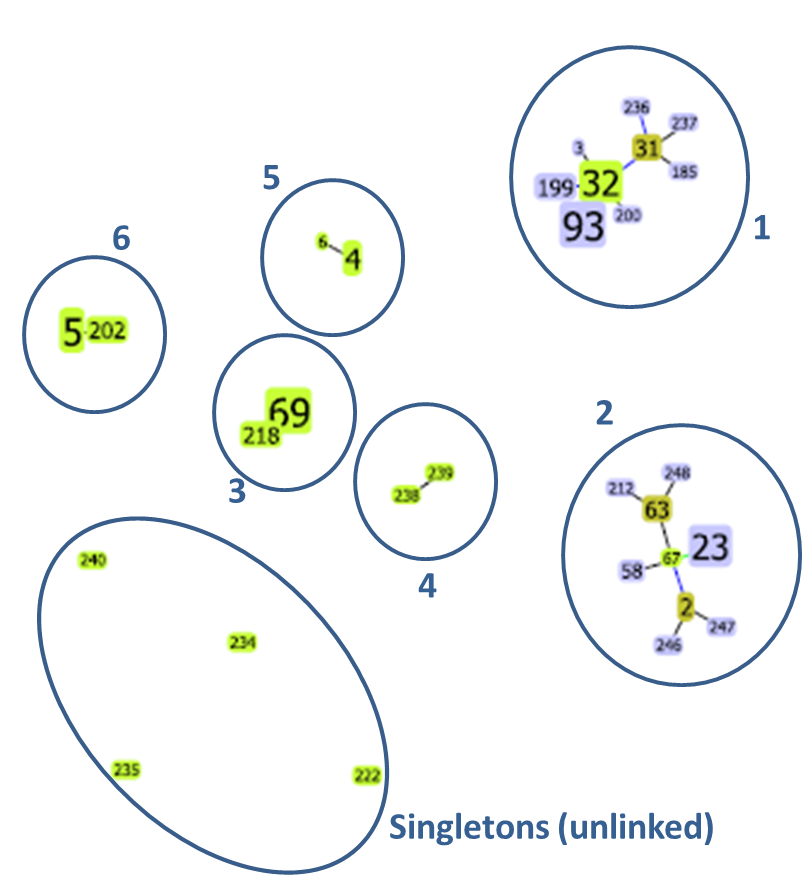


**Fig A. eBURST analysis of South African VNI isolates within dataset identified 6 unrelated clusters (here ringed and labelled 1-6), with a further 4 unrelated singleton genotypes.** Size of number in the above figure is proportional to frequency of isolates in dataset.

## Spatial Analysis

The concatenated MLST sequences were tested for spatial autocorrelation using a Mantel correlogram and a genetic variogram. No significant spatial autocorrelation could be found at α=0.05 and semivariogram modelling produced variogram models with a very short range and poor fit to the data, indicating an absence of spatial correlation between genetic and geographic distance in this study.

### Mantel Test and Correlogram

A Mantel correlogram is based on computing a Mantel test statistic (N. Mantel, Cancer Res. **27**:209-220, 1967) which compares two independently obtained distance matrices, *x* and *y*. In the application to genetic spatial structure, matrix *x* may be a matrix of genetic distances between *n* samples and matrix *y* the geographic distance between all pairs of *n* samples. Under the null hypothesis there is no correlation between genetic distance and geographic distance. The Mantel statistic is calculated by computing the correlation between *x* and *y* according to the normalized Mantel statistic,

Where *i* and *j* are row and column indices of the two similarity matrices. Values of *s* are the standard deviations of the distances in matrices *x* and *y,* respectively (P. Legendre, M.J. Fortin, Vegetatio. **80**:107-138). The correlogram is obtained by partitioning matrices *x* and *y* into discrete distance classes and computing a Mantel statistic for each partition. The test statistic for each partition is tested for significance against values calculated from random permutations of the data, with subsequent *p*-values corrected for multiple testing using the Holm method.

The Mantel statistic for the overall correlation between the genetic and geographic distance of all samples was 0.5944 (corrected *p*-value = 0.094). The simulated Mantel statistics computed from the random permutations of the data are displayed in the histogram in Fig B1 in S1 File. The Mantel correlogram is displayed in Fig B2 in S1 File. There was no significant correlation in any distance class. The lowest *p*-value obtained was 0.345.

**Fig B. Absence of strong spatial correlation with genetic distance. (1) Mantel correlogram of spatial correlation.** Each point is the value of the Mantel statistic for the given distance class. Test statistic values are plotted on the y-axis, whilst the location on the x-axis gives the mid-point for each distance class. The red abscissa represents the expectation of the Mantel statistic under no spatial autocorrelation. Values above the abscissa indicate positive correlation, whilst points below indicate negative correlation. There was no significant correlation in any distance class. (**2) Histogram of simulated Mantel statistics.** The overall Mantel statistic for the measure of correlation between the genetic distance and geographic distance was computed and is shown by the vertical black line with diamond line-ending. The significance of the returned statistic was tested by repeatedly permuting the distance matrix and calculating new test statistics. The frequency of simulated values is shown in the histogram. The probability of obtaining a test statistic at least as large as observed was *p*=0.094 and therefore no significant spatial autocorrelation was detected.

### Genetic Variograms

A fundamental tool underpinning the field of geostatistics is the semivariogram (P.J. Diggle, J.A. Tawn, R.A. Moyeed, J R Stat Soc Ser C Appl. Stat. 47:299-350; G. Matheron, Traité de géostatistique appliquée. Éditions Technip. v. p., Paris, 1962; N. Cressie, Statistics for spatial data. Wiley, New York, 1993). The empirical variogram represents the relationship between the mean semivariance for some outcome of interest, between all pairs of sample locations that are binned into discrete distance classes. Under the assumption of spatial dependence, there is a relationship between the variance of a pair of random variables and their separation distance, *d*. By fitting a model to the empirical variogram we may estimate the parameters of any spatial autocorrelation present in the data. A genetic variogram simply measures the change in variance of genetic distance between samples, instead of the variance of some continuously varying random variable. We calculated an empirical genetic variogram and used this to fit an exponential function to investigate spatial autocorrelation and this is depicted in Fig C in S1 File.

The exponential variogram model is given by:

Where γ*(d)* is the model as a function of distance, *d.* τ is the nugget variance (variance at zero separation distance, e.g. measurement error) and σ^2^ is the sill variance (the maximum variance as distance increases and spatial effects have decreasing influence). The rate at which the variogram model reaches its sill variance is controlled by the rate parameter θ. In an exponential model, the range asymptotically increases towards the sill variance, therefore an approximate range is taken as the distance at which the model variance exceeds 95% of its maximum variance (i.e. sill value). The range (ϕ) was 0.052, which is roughly a distance of 6km at the equator. The sill (σ^2^) was 4.15x10^-5^. Although the model indicated that there could be slight spatial autocorrelation up to a distance of 6km, the difference in semivariance values across the total distance range (~37km) were very small and the model did not appear to be a good fit to the data, therefore the findings were judged to not be significant.

**Fig C. Genetic variogram of South African samples.** The empty circles are the mean semivariance values between *n_l_* pairs of sample points (semivariance plotted on y-axis) in each distance bin or lag. They are located on the x-axis at the mid-point of the distance for the bin they are assigned to. The size of each circle gives the number of pairs of points in each distance class. The red line is the variogram model, which is fitted to the empirical semivariogram, assuming a nugget variance (τ) of 1x10^-5^.
